# Supplementary material for: Dietary Influences on the Longevity and Reproductive Success of Anopheles aquasalis in Laboratory Studies: Sucrose vs. Honey
Source: Insects. 2024 Dec 10;15(12):978. doi: 10.3390/insects15120978 (PMC11677520; doi:10.3390/insects15120978)
Supplement: Supplementary file 1 [file insects-15-00978-s001.zip › insects-3244838-supplementary.pdf]

Supplementary Figure S1

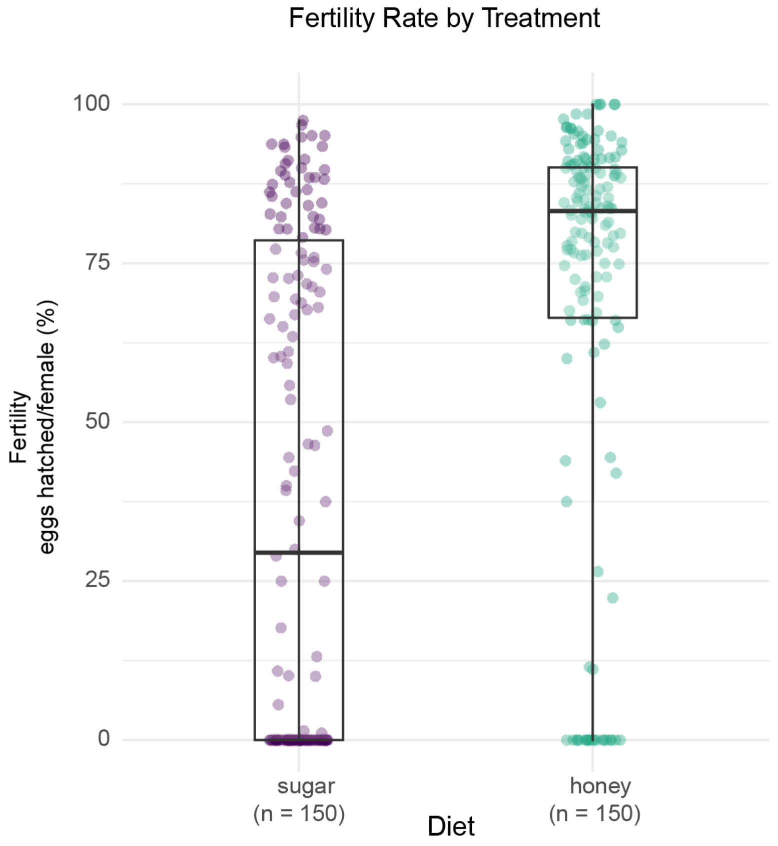

Figure S1. Effect of adult diet on percentage of hatched eggs per female (fertility) in *Anopheles aquasalis* mosquitoes. The figure shows the percentage of egg hatched for the two diet groups tested: sugar (represented by purple points) and honey (represented by green points). Each individual point represents the percentage eggs hatched from a single female. The boxes in the plot indicate the median and interquartile range for each group, with whiskers showing the data range, excluding outliers. The results of Generalized Linear Model (GLM) with Binomial Family indicate a significant difference in egg hatching rates between the sugar and honey treatments (Call:glm(formula = cbind(total_ecluded, not_ecluded) ~ treatment, family = binomial, data = fertility)

Deviance Residuals:

Estimate Std. Error z value Pr(>|z|)

(Intercept) 0.89040 0.01995 44.64 <2e-16 ***

treatmenthoney 0.59563 0.02625 22.69 <2e-16 ***
